# Two’s company, three (or more) is a simplex: Algebraic-topological tools for understanding higher-order structure in neural data

**DOI:** 10.1007/s10827-016-0608-6

**Published:** 2016-06-11

**Authors:** Chad Giusti, Robert Ghrist, Danielle S. Bassett

**Affiliations:** 1 Department of Mathematics, University of Pennsylvania, Philadelphia, PA 19104 USA; 2 Department of Bioengineering, University of Pennsylvania, Philadelphia, PA 19104 USA; 3 Department of Electrical & Systems Engineering, University of Pennsylvania, Philadelphia, PA 19104 USA

**Keywords:** Networks, Topology, Simplicial complex, Filtration

## Abstract

The language of graph theory, or network science, has proven to be an exceptional tool for addressing myriad problems in neuroscience. Yet, the use of networks is predicated on a critical simplifying assumption: that the quintessential unit of interest in a brain is a dyad – two nodes (neurons or brain regions) connected by an edge. While rarely mentioned, this fundamental assumption inherently limits the types of neural structure and function that graphs can be used to model. Here, we describe a generalization of graphs that overcomes these limitations, thereby offering a broad range of new possibilities in terms of modeling and measuring neural phenomena. Specifically, we explore the use of *simplicial complexes*: a structure developed in the field of mathematics known as algebraic topology, of increasing applicability to real data due to a rapidly growing computational toolset. We review the underlying mathematical formalism as well as the budding literature applying simplicial complexes to neural data, from electrophysiological recordings in animal models to hemodynamic fluctuations in humans. Based on the exceptional flexibility of the tools and recent ground-breaking insights into neural function, we posit that this framework has the potential to eclipse graph theory in unraveling the fundamental mysteries of cognition.

The recent development of novel imaging techniques and the acquisition of massive collections of neural data make finding new approaches to understanding neural structure a vital undertaking. Network science is rapidly becoming an ubiquitous tool for understanding the structure of complex neural systems. Encoding relationships between objects of interest using graphs (Figs. 1a–b, 4a) enables the use of a bevy of well-developed tools for structural characterization as well as inference of dynamic behavior. Over the last decade, network models have demonstrated broad utility in uncovering fundamental architectural principles (Bassett and Bullmore 2006; Bullmore and Bassett 2011) and their implications for cognition (Medaglia et al. 2015) and disease (Stam 2014). Their use has led to the development of novel diagnostic biomarkers (Stam 2014) and conceptual cognitive frameworks (Sporns 2014) that illustrate a paradigm shift in systems, cognitive, and clinical neuroscience: namely, that brain function and alteration are inherently networked phenomena.

**Fig. 1:**
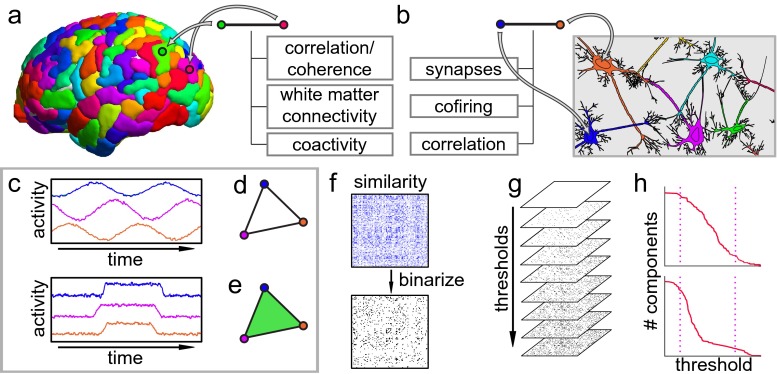
Extensions of network models provide insights into neural data. **a** Network models are increasingly common for the study of whole-brain activity. **b** Neuron-level networks have been a driving force in the adoption of network techniques in neuroscience. **c** Two potential activity traces for a trio of neural units. (*top*) Activity for a “pacemaker”-like circuit, whose elements are pairwise active in all combinations but never as a triple. (*bottom*) Activity for units driven by a common strong stimulus, thus are simultaneously coactive. **d** A network representation of the coactivity patterns for either population in **(c)**. Networks are capable of encoding only dyadic relationships, so do not capture the difference between these two populations. **e** A *simplicial complex* model is capable of encoding higher order interactions, thus distinguishing between the top and bottom panels in **(c)**. **f** A similarity measure for elements in a large neural population is encoded as a matrix, thought of as the adjacency matrix for a complete, weighted network, and binarized using some threshold to simplify quantitative analysis of the system. In the absence of complete understanding of a system, it is difficult or impossible to make a principled choice of threshold value. **g** A *filtration* of networks is obtained by thresholding at every possible entry and arranging the resulting family of networks along an axis at their threshold values. This structure discards no information from the original weighted network. **g** Graphs of the number of connected components as a function of threshold value for two networks reveals differences in their structure: (*top*) homogeneous network *versus* (*bottom*) a modular network. (*dotted lines*) Thresholding near these values would suggest inaccurately that these two networks have similar structure

All graph-based models consist of a choice of *vertices*, which represent the objects of study, and a collection of *edges*, which encode the existence of a relationship between pairs of objects (Figs. 1a–b, 4a). However, in many real systems, such *dyadic* relationships fail to accurately capture the rich nature of the system’s organization; indeed, even when the underlying structure of a system is known to be dyadic, its function is often understood to be polyadic. In large-scale neuroimaging, for example, cognitive functions appear to be performed by a distributed set of brain regions (Gazzaniga 2009) and their interactions (Medaglia et al. 2015). At a smaller scale, the spatiotemporal patterns of interactions between a few neurons is thought to underlie basic information coding (Szatmary and Izhikevich 2010) and explain alterations in neural architecture that accompany development (Feldt et al. 2011).

Drawing on techniques from the field of *algebraic topology*, we describe a mathematically well-studied generalization of graphs called *simplicial complexes* as an alternative, often preferred method for encoding non-dyadic relationships (Fig. 4). Different types of complexes can be used to encode co-firing of neurons (Curto and Itskov 2008), co-activation of brain areas (Crossley et al. 2013), and structural and functional connections between neurons or brain regions (Bullmore and Sporns 2009) (Fig. 5). After choosing the complex of interest, quantitative and theoretical tools can be used to describe, compare, and explain the statistical properties of their structure in a manner analogous to graph statistics or network diagnostics.

We then turn our attention to a method of using additional data, such as temporal processes or frequency of observations, to decompose a simplicial complex into constituent pieces, called a *filtration* of the complex (Fig. 1f–h). Filtrations reveal more detailed structure in the complex, and provide tools for understanding how that structure arises (Fig. 7). They can also be used as an alternative to thresholding a weighted complex, providing a principled approach to binarizing which retains all of the data in the original weighted complex.

In what follows, we avoid introducing technical details beyond those absolutely necessary, as they can be found elsewhere (Ghrist 2014; Nanda and Sazdanović 2014; Kozlov 2007), though we include boxed mathematical definitions of the basic terms to provide context for the interested reader. These ideas are also actively being applied in the theory of neural coding, and for details we highly recommend the recent survey (Curto 2016). Finally, although the field is progressing rapidly, we provide a brief discussion of the current state of computational methods in the AAppendix.

## Motivating examples

We begin with a pair of simple thought experiments, each of which motivates one of the tools this article surveys.

### Complexes for relationship

First, imagine a simple neural system consisting of three brain regions (or neurons) with unknown connectivity. One possible activity profile for such a population includes some sort of sequential information processing loop or “pacemaker” like circuit, where the regions activate in a rotating order (Fig. 1c, top). A second is for all three of the regions to be active simultaneously when engaged in certain computations, and otherwise quiescent or uncorrelated (Fig. 1c, bottom). In either case, an observer would find the activity of all three possible pairs of regions to be strongly correlated. Because a network can only describe dyadic relationships between population elements, any binary coactivity network constructed from such observations would necessarily be identical for both (Fig. 1d). However, a more versatile language could distinguish the two by explicitly encoding the triple coactivity pattern in the second example (Fig. 1e).

One possible solution lies in the language of *hypergraphs*, which can record any possible collection of relations. However, this degree of generality leads to a combinatorial explosion in systems of modest size. In contrast, the framework of *simplicial complexes* (Fig. 4b–d) gives a compact and computable encoding of relations between arbitrarily large subgroups of the population of interest while retaining access to a host of quantitative tools for detecting and analyzing the structure of the systems they encode. In particular, the *homology* 1 of a simplicial complex is a collection of topological features called *cycles* that one can extract from the complex (Fig. 6b). These cycles generalize the standard graph-theoretic notions of *components* and *circuits*, providing a mesoscale or global view of the structure of the system. Together, these methods provide a quantitative architecture through which to address modern questions about complex and emergent behavior in neural systems.

### Filtrations for thresholding

Second, consider a much larger neural system, consisting of several hundred units, whose activity is summarized as a correlation or coherence matrix (Fig. 1f, top). It is common practice to binarize such a matrix by thresholding it at some value, taking entries above that value to be “significant” connections, and to study the resulting, much sparser network (Fig. 1f, bottom). Selecting this significance level is problematic, particularly when the underlying system has a combination of small-scale features, some of which are noise artifacts, and some of which are critically important.

One method for working around this difficulty is to take several thresholds and study the results separately. However, this approach still discards most of the information contained in the edge weights, much of which can be of inherent value in understanding the system. We propose instead the use of *filtrations*, which record the results of every possible binarization of the network,^2^ along with the associated threshold values (Fig. 1g). Filtrations not only retain all of the information in the original weighted networks, but unfold that information into a more accessible form, allowing one to lift any measure of structure in networks (or simplicial complexes) to “second order” measures as functions of edge weight (Fig. 1h). Such functions carry information, for example, in their rate of change, where sudden phase transitions in network structure as one varies the threshold can indicate the presence of modules or rich clubs in networks (Fig. 1h). The area under such curves was used in (Giusti et al. 2015) to detect geometric structure in the activity of hippocampal neural populations (Fig. 3). Further, even more delicate information can be extracted from the filtration by tracking the *persistence* of cycles as the threshold varies (Fig. 7c).

## A growing literature

Before we proceed to an explicit discussion of the tools described above, we pause to provide a broad overview of how they have already been applied to address questions in neuroscience. The existing literature can be roughly divided into two branches:

### Describing neural coding and network properties

Because of their inherently non-pairwise nature, coactivation patterns of neurons or brain regions can be naturally encoded as *simplicial complexes*. Such techniques were first introduced in the context of hippocampal place cells in (Curto and Itskov 2008), where such an encoding was used to describe how one can reconstruct the shape of an animal’s environment from neural activity. Using the simple observa- tion that place fields corresponding to nearby locations will overlap, the authors conclude that neurons corresponding to those fields will tend to be co-active (Fig. 5b). Using the aptly (but coincidentally) named “Nerve Theorem” from alge- braic topology, one can work backward from observed coac- tivity patterns to recover the intersection pattern of the receptive fields, describing a *topological map* of the animal’s environment (Fig. 6c). Further, in order to recover the geom- etry of the environment, one can in principle introduce infor- mation regarding receptive field size (Curto and Itskov 2008). However, it seems plausible that place cells intrinsically record only these intersection patterns and rely on downstream mechanisms for interpretation of such geometry. This hypothesis was tested in the elegant experiment of (Dabaghian et al. 2014), in which place cell activity was recorded before and after deformation of segments of the legs of a U-shaped track. A geometric map would have been badly damaged by such a change in the environment, while a topological map would remain consistent, and indeed the activity is shown to be consistent across the trials. Further theoretical and computational work has explored how such topological maps might form (Dabaghian et al. 2012) and shown that theta oscillations improve such learning mechanisms (Arai et al. 2014), as well as demonstrating how one might use this understanding to decode maps of the environment from observed cell activity (Chen et al. 2014).

Even in the absence of an expected underlying collection of spatial receptive fields like those found in place cells, these tools can be employed to explore how network modules interact. In (Ellis and Klein 2014), the authors study the frequency of observation of coactivity patterns in fMRI recordings to extract fundamental computational units. Even when those regions which are coactive will change dynamically over time, cohesive functional units will appear more often than those that happen coincidentally, though *a priori* it is impossible to set a threshold for significance of such observations. Using a *filtration*, it becomes possible to make reasonable inferences regarding the underlying organization. The same approach was used in (Pirino et al. 2014), to differentiate *in vivo* cortical cell cultures into functional sub-networks under various system conditions. Finally, an extension of these ideas that includes a notion of *directedness* of information flow has been used to investigate the relationship between simulated structural and functional neural networks (Dlotko et al. 2016).

### Characterizing brain architecture or state

One of the earliest applications of algebraic topology to neural data was to the study of activity in the macaque primary visual cortex (Singh et al. 2008), where differences in the *cycles* computed from activity patterns were used to distinguish recordings of spontaneous activity from those obtained during exposure to natural images.

Cycles provide robust measures of mesoscale structures in simplicial complexes, and can be used to detect many different facets of interest in the underlying neural system. For example, in (Chung et al. 2009), the authors compute cycles that encode regions of thin cortex to differentiate human ASD subjects from controls; in (Brown and Gedeon 2012), cycles built from physical structure in afferent neuron terminals in crickets are used to understand their organization (Brown and Gedeon 2012) and in Bendich et al. (2014), the authors use two different types of cycles derived from the geometry of brain artery trees to infer age and gender in human subjects.

**Fig. 2:**
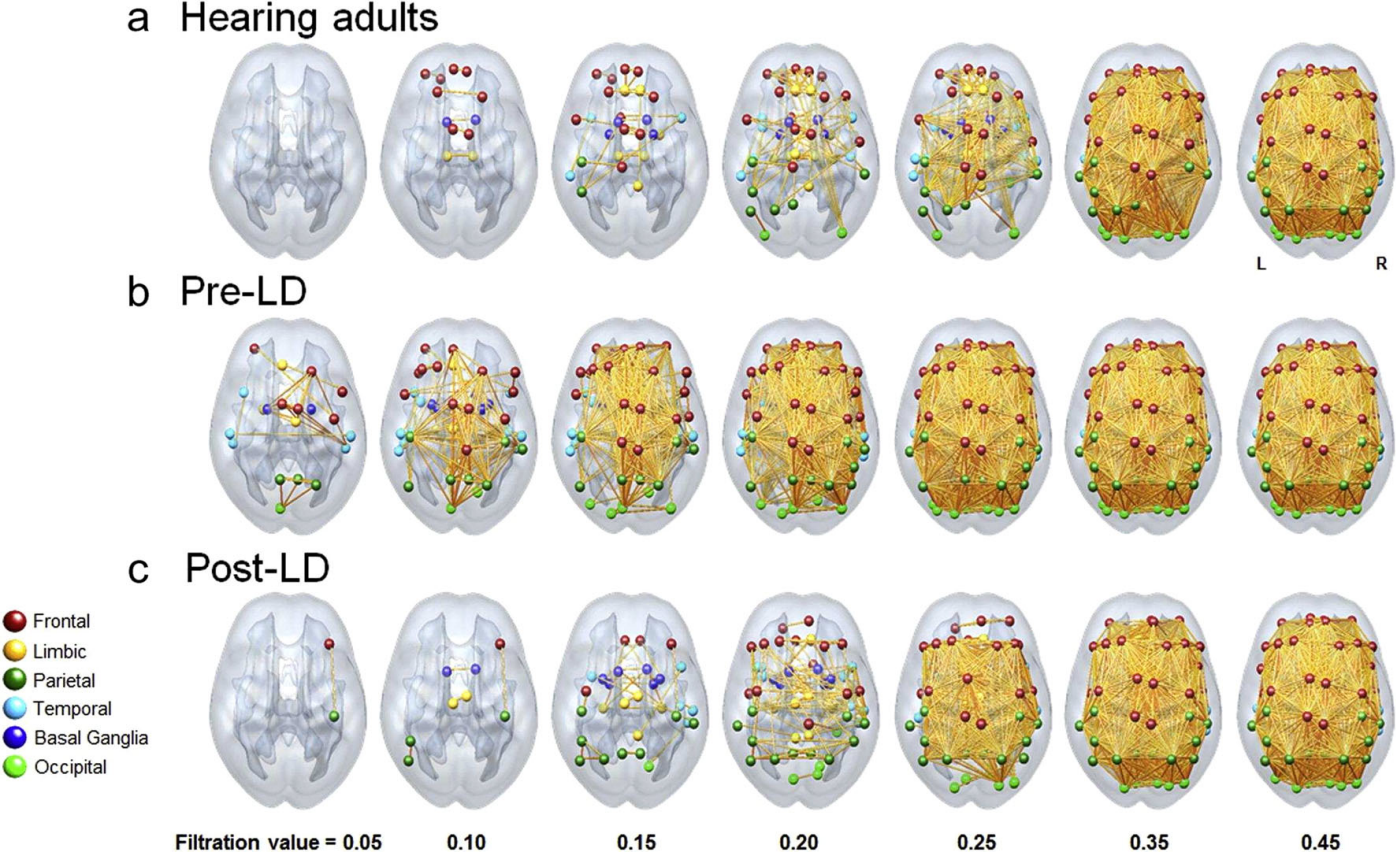
Filtered brain networks constructed from interregional correlations of density from MRI detect differences in hearing and deaf populations. Density correlation networks obtained from (**a**) hearing, (**b**) prelingual deaf, and (**c**) postlingual deaf adults. Differences in the evolution of network components across groups as the threshold parameter varies provides insight into differences in structure. It is unclear how one would select a particular threshold which readily reveals these differences without *a priori* knowledge of their presence. Figure reproduced with permission from (Kim et al. 2014)

**Fig. 3:**
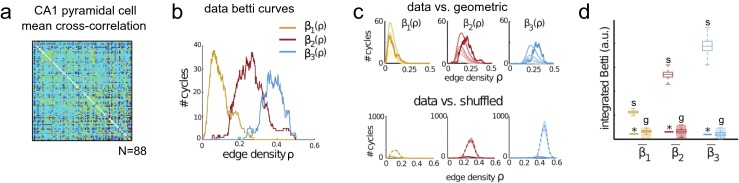
Betti numbers detect the existence of geometric organizing principles in neural population activity from rat hippocampus. **a** Mean cross correlation of N=88 rat CA1 pyramidal cells activity during spatial navigation. **b** Betti numbers as a function of graph edge density (# edges / possible # edges) for the clique complex of the pairwise correlation network in **(a)**. **c** Comparison of data Betti numbers (*thick lines*) to model random networks with (*top*) geometric weights given by decreasing distance between random points in Euclidean space and (*bottom*) with no intrinsic structure obtained by shuffling the entries of the correlation matrix. **d** Integrals of the curves from panel B show that the data (*thick bars*) lie in the geometric regime (**g**) and that the unstructured network model (s) is fundamentally different (*p*<0.001). Similar geometric organization was observed in non-spatial behaviors such as REM sleep. Figure reproduced with permission from (Giusti et al. 2015)

A common theme in neuroscience is the use of correlation of neuronal population activity as a measure of strength of the interaction among elements of the population. Such measures can be used as weightings to construct *weighted simplicial complexes*, to which one can apply a threshold analogously to thresholding in graphs. Using the language of *filtrations*, one can compute *persistence* of cycles, recording how cycles change as the thresholding parameter varies. Such measurements provide a much finer discrimination of structure than cycles at individual thresholds. The simplest case tracks how the connected components of the complex evolve; it has been used in (Lee et al. 2011) to classify pediatric ADHD, ASD and control subjects; in (Khalid et al. 2014) to differentiate mouse models of depression from controls; in (Choi et al. 2014) to differentiate epileptic rat models from controls; and in (Kim et al. 2014) to study morphological correlations in adults with hearing loss (Fig. 2). Studying more complex persistent cycles computed from fMRI recordings distinguishes subjects under psilocybin condition from controls (Petri et al. 2014), and a similar approach has been applied to the study of functional brain networks during learning (Stolz 2014). More recently, these techniques have been adapted to detect structure, such as that possessed by a network of hippocampal place cells, in the information encoded by a neural population through observations of its activity without reference to external correlates such as animal behavior (Giusti et al. 2015) (Fig. 3).

The small, budding field of topological neuroscience already offers an array of powerful new quantitative approaches for addressing the unique challenges inherent in understanding neural systems, with initial, substantial contributions. In recent years, there have been a number of innovative collaborations between mathematicians interested in applying topological methods and researchers in a variety of biological disciplines. While it is beyond the scope of this paper to enumerate these new research directions, to provide some notion of the breadth of such collaborations we include the following brief list: the discovery of new genetic markers for breast cancer survival (Nicolau et al. 2011), measurement of structure and stability of biomolecules (Gameiro et al. 2013; Xia et al. 2015), new frameworks for understanding viral evolution (Chan et al. 2013), characterization of dynamics in gene regulatory networks (Boczko et al. 2005), quantification of contagion spread in social networks (Taylor et al. 2015), characterization of structure in networks of coupled oscillators (Stolz 2014), the study of phylogenic trees (Miller et al. 2015), and the classification of dicotyledonous leaves (Katifori and Magnasco 2012). This wide-spread interest in developing new research directions is an untapped resource for empirical neuroscientists, which promises to facilitate both direct applications of existing techniques and the collaborative construction of novel tools specific to their needs.

We devote the remainder of this paper to a careful exposition of these topological techniques, highlighting specific ways they may be (or have already been) used to address questions of interest to neuroscientists.

## Mathematical framework: simplicial complexes

We begin with a short tutorial on simplicial complexes, and illustrate the similarities and differences with graphs.

Recall that a *graph* consists of a set of *vertices* and a specified collection of pairs of vertices, called *edges*. A *simplicial complex*, similarly, consists of a set of vertices, and a collection of *simplices* — finite sets of vertices. Edges are examples of very small simplices, making every graph a particularly simple simplicial complex. In general, one must satisfy the *simplex condition*, which requires that any subset of a simplex is also a simplex.

**Figure:**
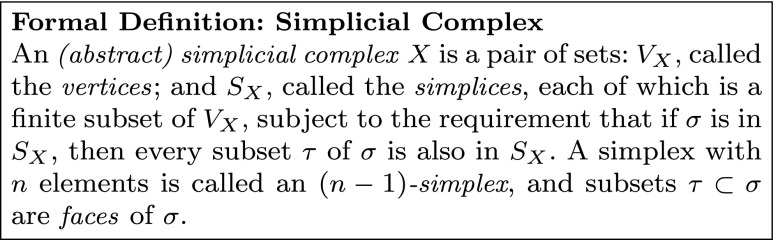

**Fig. 4:**
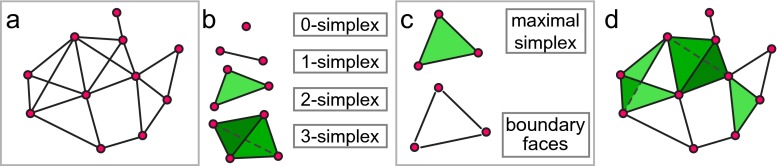
Simplicial complexes generalize network models. **a** A graph encodes elements of a neural system as vertices and dyadic relations between them as edges. **b–c** Simplicial complex terminology. A simplicial complex is made up of *vertices* and *simplices*, which are defined in terms of collections of vertices. **b** A *n*-simplex can be thought of as the convex hull of (*n*+1) vertices. **c** The *boundary* of a simplex consists of all possible subsets of its constituent vertices, called its *faces*, which are themselves required to be simplices in the complex. A simplex which is not in the boundary of any other simplex is called *maximal*. **d** A simplicial complex encodes polyadic relations through its simplices. Here, in addition to the dyadic relations specified by the edges, the complex specifies one four-vertex relation and three three-vertex relations. The omission of larger simplices where all dyadic relations are present, such as the three *bottom-left* vertices or the four *top-left* vertices, encodes structure that cannot be specified using network models

Just as one can represent a graph as a collection of points and line segments between them, one can represent the simplices in a simplicial complex as a collection of solid regions spanning vertices (Fig. 4d). Under this geometric interpretation, a single vertex is a zero-dimensional point, while an edge (two vertices) defines a one-dimensional line segment; three vertices span a two-dimensional triangle, and so on. Terminology for simplices is derived from this geometric representation: a simplex on (*n*+1) vertices is called an *n-simplex* and is viewed as spanning an *n*-dimensional region. Further, as the requisite subsets of a simplex represent regions in the geometric boundary of the simplex (Fig. 4c), these subsets of a simplex are called its *faces*.

Because any given simplex is required to “contain all of its faces”, it suffices to specify only the *maximal simplices*, those which do not appear as faces of another simplex (Fig. 4c). This dramatically reduces the amount of data necessary to specify a simplicial complex, which helps make both conceptual work and computations feasible.

In real-world systems, simplicial complexes possess richly structured patterns that can be detected and characterized using recently developed computational tools from algebraic topology (Carlsson 2009; Lum et al. 2013), just as graph theoretic tools can be used to study networks. Importantly, these tools reveal much deeper properties of the relationships between vertices than graphs, and many are constructed not only to see structure in individual simplicial complexes, but also to help one understand how two or more simplicial complexes compare or relate to one another. These capabilities naturally enable the study of complex dynamic structure in neural systems, and formalize statistical inference via comparisons to null models.

## How do we encode neural data?

To demonstrate the broad utility of this framework, we turn to describing a selection of the many types of simplicial complexes that can be constructed from data: the *clique complex*, the concurrence complex (Ellis and Klein 2014; Curto and Itskov 2008; Dowker 1952), its Dowker dual (Dowker 1952), and the independence complex (Kozlov 2007), as summarized in Table 1. In each case, we describe the relative utility in representing different types of neural data – from spike trains measured from individual neurons to BOLD activations measured from large-scale brain areas.

**Table 1:** Comparison of sample types of simplicial complexes for encoding neural data

Simplicial Complex Type	Utility
Graph	General framework for encoding dyadic relations
Clique Complex	Canonical polyadic extension of existing network models
Concurrence Complex/Dual	Relationships between two variables of interest
	e.g., time and activity, or activity in two separate regions
Independence Complex	Structure where non-membership satisfies the simplex property
	e.g., communities in a network

### Clique complex

**Fig. 5:**
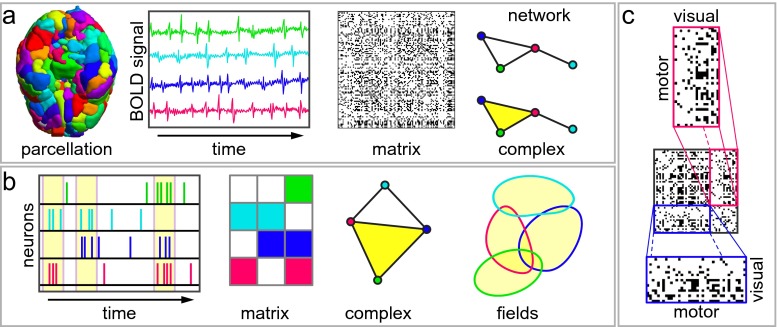
Simplicial complexes encode diverse neural data modalities. **a** Correlation or coherence matrices between regional *BOLD* time series can be encoded as a type of simplicial complex called a *clique complex*, formed by taking every complete (all-to-all) subgraph in a binarized functional connectivity matrix to be a simplex. **b** Coactivity patterns in neural recordings can be encoded as a type of simplicial complex called a *concurrence complex*. Here, we study a binary matrix in which each row corresponds to a neuron and each column corresponds to a collection of neurons that is observed to be coactive at the same time (*yellow boxes*) – i.e., a simplex. **c** Thresholded coherence between the activity patterns of motor regions and visual regions in human fMRI data during performance of a motor-visual task (Bassett et al. 2013). (*top*) We can construct a concurrence complex whose vertices are motor regions and whose simplices are families of motor regions whose activity is strongly coherent with a given visual region. (*bottom*) We can also construct a *dual* complex whose vertices are families of motor regions. The relationship between these two complexes carries a great deal of information about the system (Dowker 1952)

One straightforward method for constructing simplicial complexes begins with a graph where vertices represent neural units and edges represent structural or functional connectivity between those units (Fig. 4a–b). Given such a graph, one simply replaces every *clique* (all-to-all connected subgraph) by a simplex on the vertices participating in the clique (Fig. 5a). This procedure produces a *clique complex*, which encodes the same information as the underlying graph, but additionally completes the skeletal network to its fullest possible simplicial structure. The utility of this additional structure was recently demonstrated in the analysis of neural activity measured in rat hippocampal pyramidal cells during both spatial and non-spatial behavior (including REM sleep) (Giusti et al. 2015) (Fig. 3). In contrast to analyses using standard graph-theoretic tools, the pattern of simplices revealed the presence of geometric structure in only the information encoded in neural population activity correlations that – surprisingly – could be identified and characterized independently from the animal’s position. This application demonstrates that simplicial complexes are sensitive to organizational principles that are hidden to graph statistics, and can be used to infer parsimonious rules for information encoding in neural systems.

Clique complexes precisely encode the topological features present in a graph. However, other types of simplicial complexes can be used to represent information that *cannot* be so encoded in a graph.

### Concurrence complex

Using cofiring, coactivity, or connectivity as before, let us consider relationships between two different sets of variables. For example, we can consider (i) neurons and (ii) times, where the relationship is given by a neuron firing in a given time (Fig. 5b) (Curto and Itskov 2008); a similar framing exists for (i) brain regions and (ii) times, where the relationship is given by a brain region being active at a given time (Ellis and Klein 2014). Alternatively, we can consider (i) brain regions in the motor system and (ii) brain regions in the visual system, where the relationship is given by a motor region displaying similar BOLD activity to a visual region (Fig. 5c) (Bassett et al. 2015). In each case, we can record the patterns of relationships between the two sets of variables as a binary matrix, where the rows represent elements in one of the variables (e.g., neurons) and the columns the other (e.g., times), with non-zero entries corresponding to the row-elements in each column sharing a relation (e.g., firing together at a single time). The *concurrence complex* is formed by taking the rows of such a matrix as vertices and the columns to represent maximal simplices consisting of those vertices with non-zero entries (Dowker 1952). A particularly interesting feature of this complex is that it remains naive to coactivity patterns that do not appear, and this naivety plays an important role in its representational ability; for example, such a complex can be used to decode the geometry of an animal’s environment from observed hippocampal cell activity (Curto and Itskov 2008).

Moving to simplicial complex models provides a dramatically more flexible framework for specifying data encoding than simply generalizing graph techniques. Here we describe two related simplicial complex constructions from neural data which cannot be represented using network models.

### Dowker dual

Beginning with observations of coactivity, connection or cofiring as before, one can choose to represent neural units as simplices whose constituent vertices represent patterns of coactivity in which the unit participates. Expressing such a structure as a network would necessitate every neural unit participating in precisely two activity patterns, an unrealistic requirement, but this is straightforward in the simplicial complex formalism. Mathematically speaking, one can think of the matrix encoding this complex as the transpose of the matrix encoding the concurrence complex; such “dual” complexes are deeply related to one another, as first observed in (Dowker 1952). Critically, this formulation refocuses attention (and the output of various vertex-based statistical measures) from individual neural units to patterns of coactivity.

### Independence complex

It is sometimes the case that an observed structure does not satisfy the simplicial complex requirement that subsets of simplices are also simplices, but its complement does. One example of interest is the collection of *communities* in a network (Fortunato 2010; Porter et al. 2009): communities are subgraphs of a network whose vertices are more densely connected to one another than expected in an appropriate null model. The collection of vertices in the community is not necessarily a simplex, because removing densely connected vertices can cause a community to dissolve. Thus, community structure is well-represented as a *hypergraph* (Bassett et al. 2014), though such structures are often less natural and harder to work with than simplicial complexes. However, in this setting, one can take a simplex to be all vertices *not* in a given community. Such a simplicial complex is again essentially a concurrence complex: simply negate the binary matrix whose rows are elements of the network and columns correspond to community membership. Such a complex is called an *independence complex* (Kozlov 2007), and can be used to study properties of a system’s community structure such as dynamic flexibility (Bassett et al. 2011, 2013).

Together, these different types of complexes can be used to encode a wide variety of relationships (or lack thereof) among neural units or coactivity properties in a simple matrix that can be subsequently interrogated mathematically. This is by no means an exhaustive list of complexes of potential interest to the neuroscience community; for further examples, we recommend (Ghrist 2014; Kozlov 2007).

## How do we measure the structure of simplicial complexes?

Just as with network models, once we have effectively encoded neural data in a simplicial complex, it is necessary to find useful quantitative measurements of the resulting structure to draw conclusions about the neural system of interest. Because simplicial complexes generalize graphs, many familiar graph statistics can be extended in interesting ways to simplicial complexes. However, algebraic topology also offers a host of novel and very powerful tools that are native to the class of simplicial complexes, and cannot be naturally derived from well known graph theoretical constructs.

### Graph theoretical extensions

**Fig. 6:**
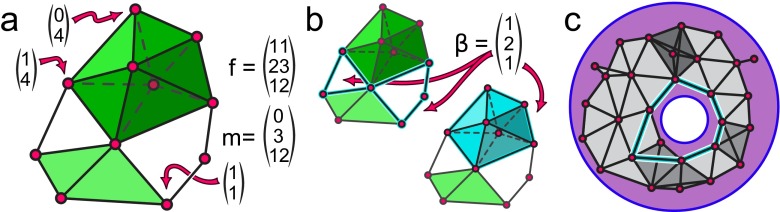
Quantifying the structure of a simplicial complex. **a** Generalizations of the degree sequence for a simplicial complex. Each vertex has a *degree* vector giving the number of maximal simplices of each degree to which it is incident. The *f-vector* gives a list of how many simplices of each degree are in the complex, and the *maximal simplex distribution* records only the number of maximal simplices of each dimension. **b** Closed cycles of dimension 1 and 2 in the complex from panel **(a)**. *(left)* There are two inequivalent 1-cycles *(cyan)* up to deformation through 2-simplices, and *(right)* a single 2-cycle *(cyan)* enclosing a 3-d volume. The Betti number vector *β* gives an enumeration of the number of *n*-cycles in the complex, here with *n*=0,1 and 2; the single 0-cycle corresponds to the single connected component of the complex. **c** Schematic representation of the reconstruction of the presence of an obstacle in an environment using a concurrence complex constructed from place cell cofiring (Curto and Itskov 2008). By choosing an appropriate cofiring threshold, based on approximate radii of place cell receptive fields, there is a single 1-cycle *(cyan)*, up to deformation through higher simplices, indicating a large gap in the receptive field coverage where the obstacle appears

First, let us consider how we can generalize familiar graph statistics to the world of simplicial complexes. The simplest local measure of structure – the *degree* of a vertex – naturally becomes a vector-measurement whose entries are the number of maximal simplices of each size in which the vertex participates (Fig. 6a). Although a direct extension of the degree, this vector is perhaps more intuitively thought of as a generalization of the *clustering coefficient* of the vertex: in this setting we can distinguish empty triangles, which represent three dyadic relations but no triple-relations, from 2-simplices which represent clusters of three vertices (and similarly for larger simplices).

Just as we can generalize the degree, we can also generalize the degree distribution. Here, the *simplex distribution* or *f-vector* is the global count of simplices by size, which provides a global picture of how tightly connected the vertices are; the *maximal simplex distribution* collects the same data for maximal faces (Fig. 6a). While these two measurements are related, their difference occurs in the complex patterns of overlap between simplices and so together they contain a great deal of structural information about the simplicial complex. Other local and global statistics such as *efficiency* and *path length* can be generalized by considering paths through simplices of some fixed size, which provides a notion of *robust connectivity* between vertices of the system (Dlotko et al. 2016); alternately, a path through general simplices can be assigned a strength coefficient depending on the size of the maximal simplices through which it passes.

### Algebraic-topological methods

Such generalizations of graph-theoretic measures are possible, and likely of significant interest to the neuroscience community, however they are not the fundamental statistics originally developed to characterize simplicial complexes. In their original context, simplicial complexes were used to study shapes, using *algebraic topology* to measure global structure. Thus, this framework also provides new and powerful ways to measure biological systems.

The most commonly used of these measurements is the *(simplicial) homology* of the complex, which is actually a sequence of measurements. The *n* ^*th*^ *homology* of a simplicial complex is the collection of *(closed) n-cycles*, which are structures formed out of *n*-simplices (Fig. 6b), up to a notion of equivalence. While the technical details are subtle, an *n*-cycle can be understood informally to be a collection of *n*-simplices that are arranged so that they have an empty geometric boundary (Fig 6b). For example, a path between a pair of distinct vertices in a graph is a collection of 1-simplices, the constituent edges, whose boundary is the pair of endpoints of the path; thus it is not a 1-cycle. However, a circuit in the graph is a collection of 1-simplices which lie end-to-end in a closed loop and thus has empty boundary; therefore, circuits in graphs are examples of 1-cycles. Similarly, an icosahedron is a collection of twenty 2-simplices which form a single closed 2-cycle.

We consider two *n*-cycles to be *equivalent* if they form the boundary of a collection of (*n*+1)-simplices. The simplest example is that the boundary of any (*n*+1)-simplex, while necessarily a cycle, is equivalent to the *trivial* *n*-cycle consisting of no simplices at all because it is “filled in” by the (*n*+1)-simplex (Fig. 4c). Further, the endpoints of any path in a graph are equivalent 0-cycles in the graph (they are precisely the boundary of the collection of edges which make up the path) and so the inequivalent 0-cycles of a graph (its 0^*th*^ *homology*) are precisely its components.

Cycles are an example of global structure arising from local structure; simplices arrayed across multiple vertices must coalesce in a particular fashion to encircle a “hole” not filled in by other simplices, and it is often the case that such a structure marks feature of interest in the system (Fig. 6c). In many settings, a powerful summary statistic is simply a count of the number of inequivalent cycles of each dimension appearing in the complex. These counts are called *Betti numbers*, and we collect them as a vector *β* (Fig. 6b).

**Figure:**
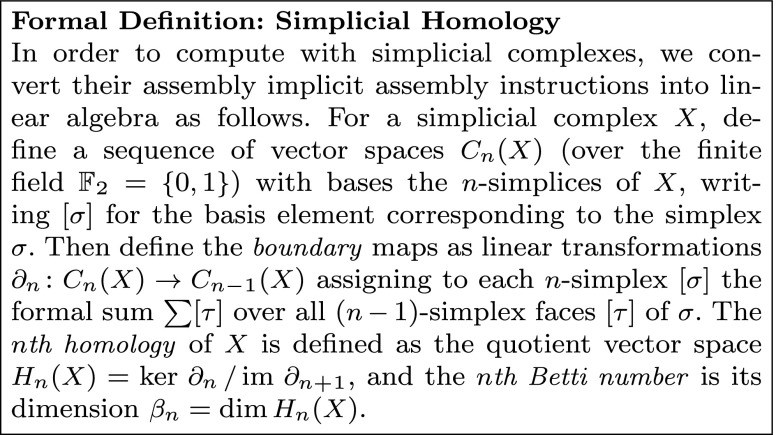

In the context of neural data, the presence of multiple homology cycles indicates potentially interesting structure whose interpretation depends on the meaning of the vertices and simplices in the complex. For example, the open triangle in the complex of Fig. 5b is a 1-cycle representing pairwise coactivity of all of the constituent neurons but a lack of triple coactivity; thus, the reconstructed receptive field model includes no corresponding triple intersection, indicating a hole or obstacle in the environment. In the context of regional coactivity in fMRI, such a 1-cycle might correspond to observation of a distributed computation that does not involve a central hub. Cycles of higher dimension are more intricate constructions, and their presence or absence can be used to detect a variety of other more complex, higher-order features.

## Filtrations: a tool to assess hierarchical and temporal structure

In previous sections we have seen how we can construct simplicial complexes from neural data and interrogate the structure in these complexes using both extensions of common graph theoretical notions and completely novel tools drawn from algebraic topology. We close the mathematical portion of this exposition by discussing a computational process that is common in algebraic topology and that directly addresses two critical needs in the neuroscience community: (i) the assessment of hierarchical structure in relational data via a principled thresholding approach, and (ii) the assessment of temporal properties of stimulation, neurodegenerative disease, and information transmission.

### Filtrations to assess hierarchical structure in weighted networks

One of the most common features of network data is a notion of *strength* or *weight* of connections between nodes. In some situations, like measurements of correlation or coherence of activity, the resulting network has edges between every pair of nodes and it is common to *threshold* the network to obtain some sparser, unweighted network whose edges correspond to “significant” connections (Achard et al. 2006). However it is difficult to make a principled choice of threshold (Ginestet et al. 2011; Bassett et al. 2012; Garrison et al. 2015; Drakesmith et al. 2015; Sala et al. 2014; Langer et al. 2013), and the resulting network discards a great deal of information. Even in the case of sparse weighted networks, many metrics of structure are defined only for the underlying unweighted network, so in order to apply the metric, the weights are discarded and this information is again lost (Rubinov and Bassett 2011). Here, we describe a technique that is commonly applied in the study of weighted simplicial complexes which does not discard any information.

**Figure:**
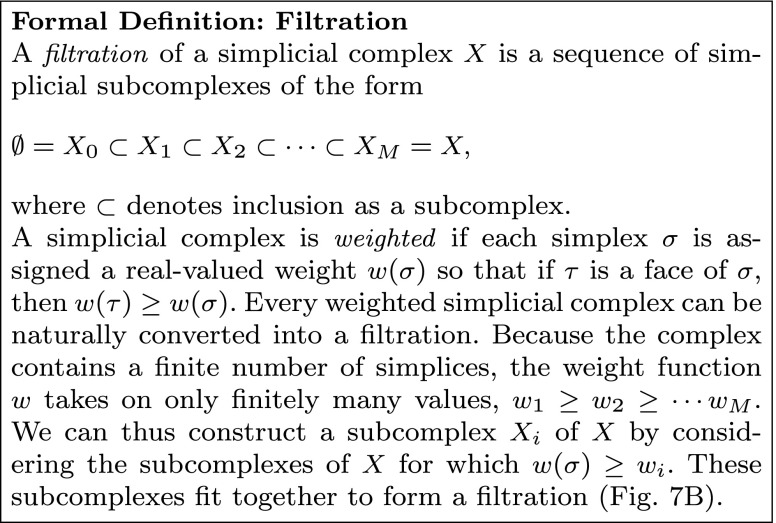

Generalizing weighted graphs, a *weighted simplicial complex* is obtained from a standard simplicial complex by assigning to each simplex (including vertices) a numeric *weight*. If we think of each simplex as recording some relationship between its vertices, then the assigned weight records the “strength” of that relationship. Recall that we require that every face of a simplex also appears in a simplicial complex; that is, every subgroup of a related population is also related. Analogously, we require that the strength of the relation in each subgroup be at least as large as that in the whole population, so the weight assigned to each simplex must be no larger than that assigned to any of its faces.

**Fig. 7:**
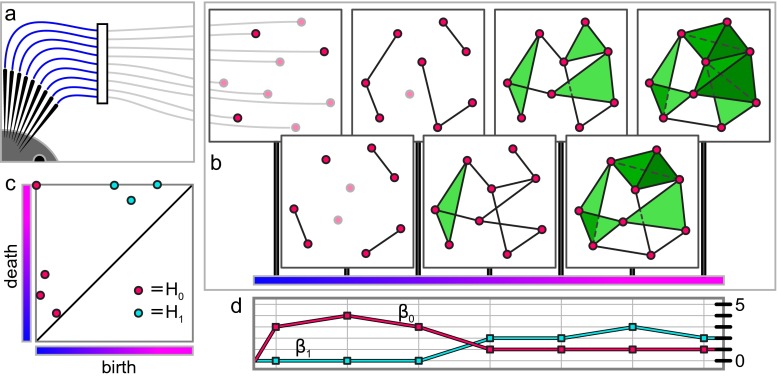
Filtrations of a weighted simplicial complex measure dynamic network properties. **a** A neural system can be stimulated in precise locations using electrical, magnetic or optogenetic methods and the resulting activity recorded. **b** A filtration of simplicial complexes is built by recording as maximal faces all patterns of coactivity observed up to a given time. A filtration can be constructed from any weighted simplicial complex by thresholding at every possible weight to produce a sequence of standard simplicial complexes, each sitting inside the next.. **c** A *persistence diagram* recording the appearance (“birth”) and disappearance or merging (“death”) of homology cycles throughout the filtration in panel **(b)**. Cycles on the *top* edge of the diagram are those that do not die. Tracking equivalent cycles through the filtration provides information about the evolution of structure as the filtration parameter changes. **d** *Betti curves* are the Betti numbers for each complex in the filtration of panel **(b)** represented as functions of time. Such curves can be constructed for any numerical measurement of the individual unweighted simplicial complexes in the filtration and provide a more complete description of structure than the individual measurements taken separately

Given a weighted simplicial complex, a *filtration* of complexes can be constructed by consecutively applying each of the weights as thresholds in turn, constructing an unweighted simplicial complex whose simplices are precisely those whose weight exceeds the threshold, and labeling each such complex by the weight at which it was binarized. The resulting sequence of complexes retains all of the information in the original weighted complex, but one can apply metrics that are undefined or difficult to compute for weighted complexes to the entire collection, thinking of the resulting values as a function parameterized by the weights of the original complex (Fig. 7d). However, it is also the case that these unweighted complexes are related to one another, and more sophisticated measurements of structure, like homology, can exploit these relations to extract much finer detail of the evolution of the complexes as the threshold varies (Fig. 7c). We note that the omni-thresholding approach utilized in constructing a filtration is a common theme among other recently developed methods for network characterization, including cost integration (Ginestet et al. 2011) and functional data analysis (Bassett et al. 2012; Ellis and Klein 2014).

The formalism described above provides a principled framework to translate a weighted graph or simplicial complex into a family of unweighted graphs or complexes that retain all information in the weighting by virtue of their relationships to one another. However, filtrations are much more generally useful: for example, they can be used to assess the dynamics of neural processes.

### Filtrations to assess temporal dynamics of neural processes in health and disease

Many of the challenges faced by cutting edge experimental techniques in the field of neuroscience are driven by the underlying difficulties implicit in assessing temporal changes in complex patterns of relationships. For example, with new optogenetics capabilities, we can stimulate single neurons or specific groups of neurons to control their function (Grosenick et al. 2015). Similarly, advanced neurotechnologies including microstimulation, transcranial magnetic stimulation, and neurofeedback enable effective control over larger swaths of cortex (Krug et al. 2015; Sulzer et al. 2013). With the advent of these technologies, it becomes imperative to develop computational tools to quantitatively characterize and assess the impact of stimulation on system function, and more broadly, to understand how the structure of a simplicial complex affects the transmission of information.

To meet this need, one can construct a different type of filtration, such as that introduced in (Taylor et al. 2015) in the context of graphs: construct a sequence of simplicial complexes with a time parameter, labeling each simplex as “on” or “off” at each time, and require that once simplices “turn on” they remain so indefinitely. If the function has the further requirement that in order for a simplex to be active, all of its faces must be as well, then a filtration is obtained by taking all active simplices at each time. Such functions are quite natural to apply to the study of the pattern of neurons or neural units that are activated following stimulation.

Interestingly, this type of filtration is also a natural way in which to probe and reason about models of neurodegenerative disease such as the recently posited *diffusion model* of fronto-temporal dementia (Raj et al. 2012; Zhou et al. 2012). Here, critical network epicenters form points of vulnerability that are effected early in the disease, and from which toxic protein species travel via a process of transneuronal spread. Indeed, these filtrations were first introduced in the context of contagion models (Taylor et al. 2015), where a simplex becomes active once sufficiently many nearby simplices are active.

### Measuring the structure of filtrations

Assuming we have encoded our data in an appropriate filtration, guided by our scientific hypothesis of interest, we might next wish to quantitatively characterize and measure the structure in those filtrations. It is important to note that any given measure of the structure of a simplicial complex can be applied to each complex in a filtration in turn, producing a function from the set of weights appearing in the complex to the set of values the measure can take (Fig. 7d). This function is a new measure of the structure of the complex which does not rely on thresholds and can highlight interesting details that would not be apparent at any fixed threshold (or small range of thresholds), as well as being more robust to perturbations in the weights than measurements of any individual complex in the filtration.

Of particular interest in this setting are those quantitative measures whose evolution can be explicitly understood in terms of the relationships between successive complexes in the filtration, as then we can exploit this framework to gain a more refined picture of the structure present in the weighted simplicial complex. Central among these in terms of current breadth of application and computability is *persistent homology*, which extends the homology of each individual complex in the filtration by tracking how cycles change as simplices are added when their weight exceeds the threshold: new cycles can form, and due to the notion of equivalence, cycles can also merge change shape, and potentially finally be filled in by larger simplices. Therefore, the sequence of complexes in the filtration is transformed by homology into an inter-related family of evolving cycles. Inside this sequence, cycles have well-defined *birth* and *death* weights, between which very complex interactions are possible. This information is often encoded in *persistence diagrams* for each degree *n* (Fig. 7c), scatter plots of birth and death weights for each cycle which give a schematic overview of how the cycles are born and die. Understanding these *persistence lifetimes* of individual cycles in the system and their statistics can provide critical information about how the system is arranged.

## Conclusion

We are at a uniquely opportune moment, in which a wealth of tools and computational methods are poised for principled development directed toward specific critical neuroscience challenges. With the feverish rise of data being collected from neural systems across species and spatial scales, mathematicians and experimental scientists must necessarily engage in deeper conversation about how meaning can be drawn from minutia. Such conversations will inevitably turn to the common understanding that it is not necessarily the individual objects of study themselves, but their relations to one another, that provide the real structure of human and animal thought. Though originally developed for entirely different purposes, the algebraic topology of simplicial complexes provides a quantitative methodology uniquely suited to address these needs.

## Appendix

### Computational aspects

The primary computational challenge in the methods here surveyed is computing the homology of a simplicial complex (or a filtration thereof). This is a significant challenge, from the view of computational complexity. The following facts are well-known (Dumas et al. 2003): the standard algorithm for computing homology involves computing the *Smith normal form* of a matrix (the boundary operator). In general, the time-complexity is doubly-exponential in the size of the matrix; it reduces to cubic complexity for simple (binary) coefficients and quadratic complexity for a sparse matrix. Worse still, the size of the relevant matrix is the total number of simplices, which grows exponentially with the dimension. This makes both space complexity (memory) and time complexity (runtime) an issue. For example, computing the persistent homology from correlation data of 100 neurons leads to a simplicial complex with approximately 10^7^ 4-simplices, while the same computation for a population of 200 neurons involves two orders of magnitude more.

However, there are a number of ways to exploit the structure inherent in simplicial complexes to mitigate this combinatorial growth in complexity. One effective approach is to use preprocessing to collapse the size of the complex – sometimes dramatically – without changing the homology. This is the basis of various reduction algorithms: see, e.g., (Kaczynski et al. 2004; Mischaikow and Nanda 2013). Another approach is to use the fact that homology can be computed locally and then aggregated, allowing for distributed computation over multiple processors and memory cores (Bauer et al. 2014). Finally, computing *approximate* homology further reduces complexity in difficult cases while still providing useful statistical information (Sheehy 2013).

A comprehensive survey on the state-of-the-art in homology software with benchmarks as of late 2015 appears in (Otter et al. 2015). Because algorithmic computations of topological quantities is a relatively recent innovation and because the field is now being driven by accelerating interest in the broader scientific community, it is our expectation that new ideas and better software implementations will dramatically improve our ability to perform these computations over the next few years. Further, as is commonly the case, computations on “organic” data outperform the worst-case expectations for the algorithms; in practice, the difficulty of homology computation in a particular dimension tends to grow linearly in the number of simplices in that dimension. Thus, we are optimistic that the computational tools necessary to apply these ideas to neural data will be available to meet the needs of the neuroscience community as they arise.

## Footnotes

1 Names of topological objects have a seemingly pathological tendency to conflict with terms in biology, so long have the two subjects been separated. Mathematical homology has no *a priori* relationship to the usual biological notion of homology.

2 Or some suitably dense subset of the binarizations, in the case of very large systems.
